# Patient and staff experiences of a community-based diagnostic clinic for chronic eye conditions: Qualitative analysis

**DOI:** 10.1038/s41433-026-04528-8

**Published:** 2026-05-19

**Authors:** Angus I. G. Ramsay, Joy Adesanya, Juan Carlos Bazo-Alvarez, Helen Baker, Jocelyn Cammack, Caroline S. Clarke, Holly Elphinstone, German Alarcon Garavito, Sir Peng Tee Khaw, Stephanie Kumpunen, Grant Mills, Dhakshi Muhundhakumar, Steve Napier, Siyabonga Ndwandwe, Peter Scully, Anne Symons, Hari Jayaram, Sobha Sivaprasad, Paul J. Foster, Naomi J. Fulop

**Affiliations:** 1 Research Department of Behavioural Science and Health, University College London (UCL), London, UK; 2 Moorfields Eye Hospital NHS Foundation Trust, London, UK; 3 Research Department of Primary Care and Population Health, University College London (UCL), London, UK; 4 Escuela de Psicología, Universidad Cesar Vallejo, Trujillo, Peru; 5 National Institute for Health and Care Research (NIHR) Biomedical Research Centre (BRC) at Moorfields Eye Hospital NHS Foundation Trust and UCL Institute of Ophthalmology, London, UK; 6 UCL Institute of Ophthalmology, London, UK; 7 The Bartlett School of Sustainable Construction, UCL, London, UK

**Keywords:** Health services, Health care

## Abstract

**Background:**

In the UK, ophthalmology appointment delays are a longstanding issue, with serious implications for population health, and were exacerbated by the COVID-19 pandemic. In response to these pressures, a major eye hospital organisation established a community-based clinic providing testing for stable glaucoma and medical retina patients in a London shopping centre. This clinic reduced appointment delays significantly. However, little is known about how patients and staff experience services of this kind.

**Objectives:**

To understand experiences of patients attending and staff working in this clinic.

**Method:**

Semi-structured interviews with staff (*n* = 29) and patients (*n* = 9). Observations of service delivery (*n* = 6). Data were analysed thematically.

**Results:**

Four themes were identified: (1) delivering eye appointments; (2) clinic setting; (3) communication and engagement; and (4) future of diagnostic clinics. Patients were overwhelmingly positive about their experiences of the clinic, citing shorter appointment lengths and supportive, friendly technicians. Some patients found the clinic harder to reach than their local hospital, but noted this would not prevent future attendance. Technicians enjoyed working at the clinic and felt valued, whilst describing challenges in communicating with offsite clinicians and concerns about professional development. Staff and patients supported future use of services of this kind.

**Conclusions:**

Patients and staff experienced this community-based eye clinic very positively. This, alongside the clinic’s impact on appointment delays, suggests that such clinics may be valuable in future. Services of this kind may be optimised and sustained in future by enhancing pre- and post-appointment patient communication, providing transport solutions, and strengthening technician career development.

## Background

Missed or delayed appointments are a leading cause of avoidable blindness in patients with chronic eye disease [1]. In the UK, appointments to monitor and diagnose chronic eye conditions have traditionally been conducted in hospital clinics delivered through the National Health Service (NHS), a publicly-funded health system that organises and provides comprehensive healthcare, including primary care and hospital care [2]. With over 9 million appointments per year, ophthalmology is the most active outpatient specialty in the English NHS [3]. The backlog in diagnostic and monitoring appointments for chronic eye conditions in England is longstanding, and was exacerbated by the COVID-19 pandemic [4]. Demand for these services is anticipated to grow in England, given that an ageing population is associated with increased incidence of chronic eye disease [3, 5]. During the pandemic, appointments for chronic eye conditions were paused or delayed to reduce the risk of contagion [6]. This contributed to further increases in assessment and interventions for patients with stable/non-acute conditions [6]. This is part of a broader concern: waiting list targets across the English National Health Service (NHS) have been missed consistently for many years, contributing to hundreds of people suffering avoidable sight loss [7].

One policy response to these challenges has been the drive to shift care from hospitals into communities: in particular, the NHS ‘10 year plan’ sets out proposals to introduce neighbourhood healthcare tailored to the population they serve; this is anticipated to increase accessibility and support improved patient engagement with services, thus contributing to improved public health and reduced pressure on hospital services [8]. For example, in 2020, NHS England proposed the widespread roll-out of diagnostic hubs, clinics that provide community-based diagnostic services [9]. Such clinics may enhance engagement with services (especially in underserved communities) and potentially reduce backlogs [10]. However, a review of the international evidence found little research on community-based diagnostic clinics, with only five studies conducted in the UK [11] and little evidence of such clinics bringing services closer to patients [12]. In addition, the two other policy responses – analogue to digital and prevention of disease (disability) – also align with the creation of community-based ophthalmic clinics. Digital change is a key requirement of community ophthalmology virtual clinics aligned with the concept of detecting changes (ocular and general health) to prevent disability before it manifests [13].

In ophthalmology, ‘virtual clinics’ – combining technician-delivered diagnostic testing and asynchronous specialist clinician review – have been established for many years in the UK [13–16]. These clinics deliver testing that is comparable to hospital diagnostics, and are overall acceptable to patients [16–19]. Services of this kind may be well-suited to community settings.

In September 2021, in response to the COVID-19 pandemic, a large London-based eye hospital established a research-focused community-based clinic to provide diagnostic monitoring of stable glaucoma and medical retinal patients. The clinic had two main aims: (1) to address the growing delays in diagnostic appointments for patients with stable eye conditions; (2) to test different configurations of diagnostic instruments and assessment delivery (see ‘Setting’, below). Attractions of placing the clinic in a community setting (as opposed to an NHS hospital setting) included greater accessibility (e.g. additional public transport routes and car-parking options), far fewer delays in planning for building/utilities (permitting rapid expansion of capacity) and greater proximity to community pharmacy (permitting faster turnaround of prescriptions than in hospital settings). Introduction of this clinic was associated with significant reductions in appointment delays across the hospital organisation’s network, demonstrating potential benefits of such services in this context [20].

Evidence suggests that good patient experience increases attendance for follow-up appointments and adherence to care plans, resulting in better patient outcomes [21, 22]. Positive staff experience, e.g. around engagement and wellbeing, is associated with better service performance, workforce retention, and patient satisfaction [23, 24]. However, there is little evidence on how community-based diagnostic services are experienced by staff working in and patients attending these services [11, 25].

To address these important gaps, this study used qualitative methods to analyse how patients and staff experienced service delivery in this diagnostic monitoring clinic, addressing the following questions:

- What were patients’ experiences of attending the community diagnostic clinic?
- How did staff experience delivering care in the community diagnostic clinic?

## Method

### Setting

A Specialist Eye Hospital NHS organisation in London provided all the studied services. The intervention site was a community-based diagnostic clinic, which monitored patients with stable glaucoma or chronic medical retina (MR) conditions, e.g. diabetic retinopathy. Patients were registered with hospitals (EH1, EH2 and EH3) which were located across North West London within a 10-mile radius of the clinic. Patients were referred to the clinic by their local hospital, who sent patients a referral letter to inform them of their appointment and share additional information about the clinic. The clinic was situated in a retail unit in a shopping centre. Diagnostic equipment included Humphrey Automated Perimeters (HFA3) and Optical Coherence Tomography Devices (Cirrus OCT 6000) from Zeiss, Optical Coherence Tomography Devices (Spectralis) from Heidelberg Engineering and Ultra-Widefield Retinal Imaging Devices (California *rg*) from Optos. It was staffed by non-clinical diagnostic technicians (recruited predominantly from a range of non-healthcare sectors, e.g. retail and hospitality, to support delivery of this clinic) who undertook all diagnostic tests; resulting images were uploaded to a secure hospital server for asynchronous review by ophthalmic specialists, with results shared with patients by letter. The clinic aimed to provide services for patients with stable, non-complex conditions, allowing hospital specialist resources to be devoted to patients with unstable or urgent conditions, and with frailty and mobility issues. As part of the intervention, the clinic tested different layouts of equipment in iterations. Key arrangements of equipment included ‘lanes’, where each instrument in a testing menu was situated one after another, and patients moved from one testing-station to the next in a straight line; and ‘clusters’, where two or more types of machine were co-located in the same space. In each iteration, combinations of these arrangements were adapted to take account of staff and patient feedback. The aim of testing different layouts was to identify ways to optimise the clinic’s efficiency for staff and patients, including safely maximising throughput and minimising bottlenecks and waiting time for equipment.

### Design

Semi-structured interviews were conducted by two researchers (JM, AIGR) between December 2021 and February 2023, as the clinic continued to develop.

### Sample

Interviewees included patients who attended the clinic. We sampled patients to achieve diversity across condition (glaucoma and medical retinopathy), sex, and age (Table 1). We sampled staff who worked at the clinic (technicians and administrators of different seniority), and staff working at eye hospital sites that (1) referred patients to the clinic and (2) provided specialist review of scans (EH1, EH2, and EH3) (Table 1). This approach allowed us to explore perspectives from staff at different referring sites, some of which were more distant from the clinic.

**Table 1:** Data analysed in this study.

Data	Clinic	EH1	EH2	EH3	*Total*
*Interviews*					
Staff					
Technicians	15	-	1	1	***17***
Administrative staff	5	2	1	1	***9***
Senior clinic staff	3	-	-	-	***3***
Clinicians	-	5	1	-	***6***
Patients	9	-	-	-	***9***
Age ^a^	Mean=64.75 years (range 51-72 years)				
Sex	Female=5; Male=4				
Condition	Glaucoma=4; Medical retina=5				
*Observations*	6	-	-	-	***6***
***Total***	***38***	***7***	***3***	***2***	***50***

^a^ Ages provided only by 6 patients.

### Recruitment and consent

All patients were invited to participate in research related to the clinic in advance of their appointment. Potential patient interviewees were drawn from patients who had consented to this. Potential patient interviewees were identified by clinic staff and contact details were shared with the researchers. Potential staff interviewees were identified through engagement with service leads and service observations.

Recruitment and consent documents are provided in Supplementary File 2. For all potential interviewees, researchers shared an information sheet and consent form for interviewees to consider. These forms explained what taking part in an interview would involve and how data would be used. If interviewees were happy to take part, a date for interview was arranged. Before the interview commenced, the researcher ran through the consent form with the interviewee; interviewees’ verbal consent was recorded by the researcher and confirmation of consent was sent to the interviewee. For non-participant observations of activities related to service delivery and information sheet was shared with members of staff and discussed at team meetings in advance of observations taking place. Staff provided written consent to be observed.

### Data collection

Interviews were conducted by researchers JM and AIGR using videoconferencing (staff, using MS Teams or Zoom) or telephone (patients). Both JM and AIGR were health services researchers with backgrounds in psychology and experience of conducting qualitative and mixed methods research. The staff interview topic guides covered experiences of working in and delivering diagnostic services in the clinic; the patient interview topic guide covered experiences of attending the clinic and standard eye appointments (Supplementary File 2). Saturation was achieved when researchers agreed in review meetings that consistency in themes and views was being seen in interviews [26]. Observations of service delivery in the clinic (patient-provider interactions; use of space and equipment; patient flow) were conducted and analysed. Each observation lasted 3–5 h and was recorded as written fieldnotes (*n* = 6) (Table 1).

### Analysis

Interviews were professionally transcribed, and transcripts were managed in NVivo [27]. Data were analysed thematically [28]. First, themes were identified inductively from a sample of the data (by AIGR, JM); data were then coded by one researcher (JM), with ongoing review by a second researcher (AIGR) to support further development of coding (including reviewing the coding of 20% of transcripts). Interpretation of findings was discussed regularly with other team members (JM, AIGR, NJF, JM, CSC, SNa, SNd); emerging findings were discussed with the wider author group at regular team meetings.

### Patient and public involvement and engagement (PPIE)

PPIE was central to this study: co-author SNa was part of the project team, discussing all aspects of research in project meetings and providing written feedback on relevant research documents. Through this approach, SNa contributed to project management, study design (including research tools and recruitment documentation), interpretation of findings, and co-authoring of the article. Co-authors HB and JC are PPIE specialists in the context of eyecare, and contributed to study design, interpretation of findings, and co-authoring of the article.

### Ethical approval

This study was approved by the North East-York Research Ethics Committee (REC reference 21/NE/0164) on 17^th^ August 2021.

## Results

**Fig. 1:**
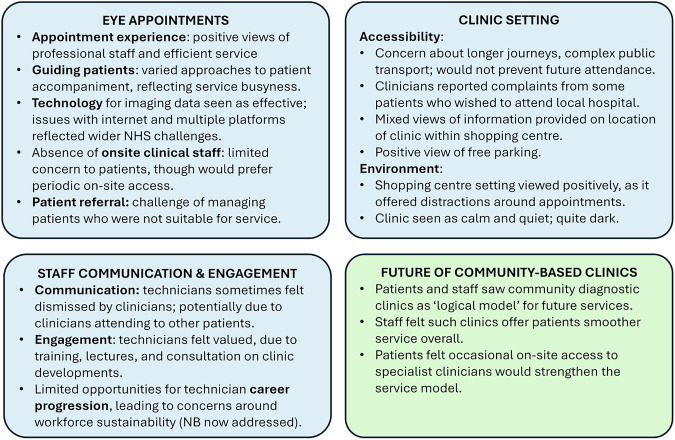
Overview of themes and findings.

The analysis generated four main themes (see Fig. 1 and Table 2): (1) delivering eye appointments; (2) clinic setting; (3) communication and engagement; and (4) future of these clinics.

**Table 2:** Illustrative quotes.

Theme	Quote
**Theme 1: Delivering eye appointments**	
1.1 Appointment experience	“Yes, I mean it didn’t sort of waste time as in like the waiting time was a lot less because I’ve been to [EH2] before and it’s taken like a couple of hours and here it was like within an hour everything was done if not less than hour. So yes it was good in that way.” (Patient 03) “The place itself had a somewhat industrial feel, but that’s neither here nor there. Once we got going it was fine” (Patient 07) “A lot quicker than if I was in a main hospital. It was a lot quicker, a lot smoother.” (Patient 06) “They [*technicians*] were lovely. They were professional but they were lovely. As in the two are not mutually exclusive, you know what I mean” (Patient 06) “They [*technicians*] were messing around and trying to keep you at ease. They were just trying to keep your spirits up basically while you’re going through this whole process.“ (Patient 05)
1.2 Referral	“When you’re looking at this patient, you don’t know people’s mobility or anything like that […] So again, when they were pulling people over, perhaps they didn’t know, oh, actually that patient’s a transport patient. That patient’s got mobility issues or whatever. Again that was more at the beginning. Nowadays they don’t tend to take so many from us. We tend to book ourselves into their clinics, that sort of - Where that mishap led to was then, okay, it becomes now our responsibility to go, “Okay, we can book certain patients into their clinics,” and then we know from which clinics to take, which patients to take and put into their clinics” (External staff 05 – coordinator) “One of the challenges that remains, is using our hospital information system to select people with a suitable, we still do get people turning up at Brent Cross who are blind in both eyes and really frail and they should be in a hospital rather than in a shopping centre” (Staff 10 – consultant ophthalmologist) “We had a technician who was double checking the patient notes after the patients are gone. So the following day we would have a technician who will go over all the patients that were seen making sure that everyone was referred to the right clinic and there was nothing missed out” (Staff 15 – senior technician)
1.3 Guiding patients	“I was led by the hand around all of the stations that I was needed to by somebody that was looking after me. I was led from station to station and handed over, they introduced themselves, they were very polite, and it all went swimmingly.” (Patient 01) “It depends on the day-to-day operation, of course, if we don’t have the sufficient amount of technicians, what we try to do is allocate each technician to a specific station. […] If we have sufficient amounts, full capacity of technicians, then each technician takes each patient all the way through from A to B basically (Staff 03 – technician) “last Saturday, had a 90 year old patient come in, very fragile lady, very old, walking on a walking stick, she could barely walk, her son was you know guiding her all the way, holding her, sitting her down, picking her up and stuff like that we didn’t have a wheelchair so we had to go all the way to the [shopping centre] mobility park, get a wheelchair bring it in, get them, then do that and then go drop the wheelchair all the way back” (Staff 02 – technician)
1.4 Technology	“the ultra-wide band often has a glitch […], we had a couple of days ago one of the testing devices, software systems that the whole of [clinics’] support system went offline for a day and that happens periodically with each of the different bits of kit that’s linked up to a computer.” (clinic staff 10 – technician) “Of course, there’s been pictures where the software’s been a problem so assessments couldn’t take place, imaging couldn’t take place. There’s been issues with the patients not having the scan because they’ve not, for some reason missed the scan, then there’s been issues with us accessing the images because the system’s down. But it would be great if the images were all on the same platform rather than opening three different imaging servers but again that is a problem when you’ve got multiple vendors and imaging” (External staff 23 – consultant ophthalmologist)
1.5 Clinical staff onsite	“I was fairly happy, the only thing is when you, when I used to go the hospital because you had the doctors there, they’d give you the feedback straight away. So they showed you exactly what’s wrong and everything and you can see the stuff. So that’s probably the only drawback I think with [Hub1].” (Patient 03) “I prefer going you know to [*local eye hospital*] and they can explain things to me there and then” (Patient 04) “Because I was only going for routine screening, I didn’t have actually have any issues that I was aware of that needed – I am surprised that they didn’t at least have one doctor onsite just in case anybody needed something” (Patient 06) “the other thing is in [local eye hospital], doctors are available there. They see me and they decide on the next step. Here it is done through the communication by letters. Probably within two weeks but in [local eye hospital], doctors say just after the test.” (Patient 09)
**Theme 2: Setting of the clinic**	
2.1: Accessibility	Patients: “I normally drive but normally I would have gone to [EH2], it’s just a straight run on the bus from my home. And on this particular instance I had to go into [area] and then change buses and the only bus I could get went round the houses. It took me I think over an hour and a half to get to [clinic] on the bus.” (Patient 07) “Getting to the shopping centre was great, especially as the parking in that one is free. Any other shopping centre would have charged me” (Patient 01) “I didn’t really want to travel that far because if they have to put drops in my eyes I wouldn’t be able to get home that easily” (Patient 04) “But what I believe that when the eye tests are being done it really has to be where public transport is easily available. And I think from where I stay it is quite difficult to get to [Clinic]. I tell you that I think one of the important demographic situations you need or we need all is that easy access to both underground and bus. Here your underground station is quite far and the bus is from my place, it usually goes around quite a lot. And since most of that test is going to be dilated, I can’t use my own car, so there is slight problem from where I live to get to your place.” (Patient 09) [On why the patient got someone to transport them] “Buses wasn’t really, about three to four buses - and if I missed one the timing wasn’t right either“ (Patient 08) Staff: “location […] has been a bit of a tricky issue especially for some of our older patients mainly because that area is generally best accessed by car. And the catchment of patients that we send to that area live right next to the previous eye clinic which would have been external hospital 2 or [external hospital 3] and for them it’s perceived as a big adjustment. So, I think for very old patients that tend to be the demographic of glaucoma clinics, unless they have transport or help it becomes more challenging” (Clinic staff 20) “We were also told that [clinic] would make our lives easier. I cannot tell you how that is a lie, a total lie, it’s just been a nightmare. Some days it’s [clinic], [clinic], every day on the phone. Complaints from PALS, they all come to us. They don’t go to [clinic], they come to us because there are patients that are refusing [clinic]” (External staff 02, EH2) “They don’t want to go there, you know, ‘Why should I go over there? You are sending me away, you’re discharging me, you’re not going to see me anymore,’ that kind of concern. So the doctors they actually, when they call them, they reassure them. They say, ‘You’re not going away: you are still under our control, you are still under [hospital]’”(External staff 03, EH2) “And what we will do, if they absolutely will not go, we will cancel. I’m just keeping a list of people and I’m fitting them in where I can. If the doctor get annoyed and have a go at us here about doing it I don’t care, because at least the patient is still getting their eye care. Because some people were at the point where they were saying, I would rather go blind than go.” (External staff 01, EH2)
2.2 Environment	Patients: “You can just walk around the centre for half an hour. You can just pop over and get something to eat, you can walk around and go for a few shops, you’ll be back in no time.” (Patient 05) “I would say it’s quite calm, it was not very busy. At [EH2] we have many times have much more people at the same time so hub is fine. I would say it’s quiet compared to [EH2] here.” (Patient 09) “Only one comment on the whole environment, that when I transited, when I entered from the shopping centre mall into what would be the store, into the premises, it was really very bright outside and it was quite dim inside, which I totally understand but it did take a little bit to get used to.” (Patient 01) Staff: “They walk in through a dim lit area, they think it’s all going to be quite dingy and closed in but they end up spending the rest of that time saying, ‘Oh, this is really nice and big and bright and airy.’” (clinic staff 05 – technician) “We are having a lot of follow-up patients, who come every two or three months […] They are happy with the whole shopping centre environment because if they’re coming with a family member they know they can park for free, of course, the family member can go and do a bit of shopping here and there and then by the time they’ve finished within the hour or so, they can come back, so, they’re not waiting around” (clinic staff 03 – technician) “There is a lot of space now to put the patient into the VF *(visual field)* section, it’s a wide open plan just two specific bays which we can get a patient who has mobility issues or a wheelchair to come through” (clinic staff 03 – technician)
**Theme 3: Staff communication, engagement, and progress**	
3.2 Communication between technicians and clinicians	“Because sometimes, you know, we are being dealt with in a rude manner on the phone, when we call to ask for assistance. Bear in mind, we don’t have that great knowledge behind whatever issues we come across when we do the scans. This is why we are calling to ask these others for help. But they are not forthcoming, some of them are not forthcoming.” (Clinic staff 11 - Technician) “So the problem I guess is, that’s what I mean about the coordination and organisation of it which I’ve helped but we don’t have the right infrastructure. So the hope we have is we have a coordinator to make sure that people are staffed accordingly, so every session has a clinician on site, we’ve asked for that. And the reason this is negative response to the team in [Hub1] is some days the doctors are then pulled out to cover face to face clinic and there’s no doctor assigned and it’d be diverted to the busy face to face clinics downstairs.” (Staff 23 – consultant ophthalmologist)
3.3 Staff engagement	“And I’ve had a couple bits of feedback direct from doctors, which has given me a little bit more confidence in what I’m doing. And it also gives me a little bit more of a lust to want to go and learn about certain conditions in the eye and why things look the way they do. So, the education side of things I’m being sparked to want to go and get into it more, which is good.” (Clinic staff 05 – Technician) “I think, one of the greatest things that I’ve been able to do, you know, for me is build a team that really respects each other that they enjoy their working environment and that’s like, one of the core things that I love, that, you know, [they] allowed us to be like, ‘okay, what do you want as staff members’, you know? ‘What is a good working environment, for you? How do we include you in the decisions you make here?’.” (Clinica staff 18 –coordinator) “both Iterations I was there on my days off coming here and you know, putting in my experience and letting them know what would work best in what way. Where the machines should be, where they should be facing how the patients should be coming in. I mean a lot of that has, you know, been used a lot of my ideas have been used” (Clinic staff 02 – technician)
3.4 Career progression	“I mean for people with children and that especially, it was a bit worrying because no one really was sure whether… Like we weren’t sure whether we’d have a permanent role.” (Clinic staff 12 - technician) “it’s not really a promotion, you’re not a team leader, you’re not a supervisor and neither you’re a senior. So in that way I’m not sure. I would like to be considered for promotion of course, that’s one of my main objectives” (Clinic staff 02 – technician)
**Theme 4: Future of diagnostic hubs**	
4.1 Future of hubs	“I think it’s needed. We’re in new age so I think it’s needed to go forward with the times and really to understand that there’s people who are busy every day and if we’re going to be using Central London as a hub for everything it’s just not going to work.” (Patient 05) “You know, it’s all about service, and I feel it’s working. A lot of patients do say the same thing as well. They prefer it this way.” (Clinic staff 11 – Technician) “I think without them [hubs] we’re not going to be able to deal with the, I mean glaucoma it’s only going to increase the number of patients that we’re seeing. And you never really discharge a glaucoma patient from your clinic so, the number just goes up like exponentially. And it’s just inconceivable that we would then see those patients, all of them in face to face clinics, it’s not practical, it’s risky for the patients. And the benefit of the virtual clinics is that you can get a lot more information a lot faster and you can actually monitor your patients a lot more closely.” (External staff 20 – clinician)

### Delivering eye appointments

#### Appointment experience (Table 2, Section 1.1)

Patients were overwhelmingly positive about their clinic experiences, reporting that their appointments took less time and involved less waiting than in traditional services. One patient stated their experience felt rushed and ‘industrial’ (though did not see this as a major issue). Still, others valued being seen quickly and described their time in the clinic as efficient rather than stressful. Several patients referred to the friendly and professional approach adopted by technicians. Most also felt that the clinic’s efficiency compensated for the extended journeys.

#### Patient referral (Table 2, Section 1.2)

The diagnostic clinic was conceived to provide services for patients considered to have stable, non-complex conditions and no significant mobility issues, on the basis that patients with complex conditions or frailty are better provided for in hospital environments. Clinic staff felt that most patients attending had been appropriately assigned to the clinic. Occasionally, unsuitable patients were allocated to the clinic due to staff having insufficient information about the patients’ physical condition, or occasional administrative errors (e.g. referring patients who had mobility or vision issues that made them ineligible for services). Initially, the clinic was not organised to accommodate such patients, e.g. wheelchairs were unavailable. Consequently, staff (sometimes with help from the patient’s friends or family) had to help patients move around the clinic. Several staff members described how the first iteration layout made this difficult, as some equipment had been positioned far apart. Subsequently, wheelchairs were made available, although some staff felt this still caused a problem in Iteration 2 because the equipment positioning made manoeuvring wheelchairs difficult. These issues were felt to have been largely addressed in the third layout iteration, following feedback from staff – although it remains the case a relatively small minority of people with frailty/mobility issues are allocated to hospitals, as they have the resources necessary to provide safe care.

#### Guiding patients (Table 2, Section 1.3)

During observations, it was noted that staff would sometimes stay 1-to-1 with a patient and conduct every test with them (in line with recommendations to minimise interpersonal contacts during the Covid-19 pandemic); at other times, technicians remained at one imaging station and patients moved from station to station. In the first scenario, patients were called from the waiting area by a technician who introduced themselves and undertook initial tests. The technician explained the tests and the asynchronous review and management planning. Upon completion of each test, the technician would accompany the patient to the next instrument, explain the test, and guide them through the procedure, e.g. ensuring correct positioning, until all tests were completed. Alternatively, the technician undertaking the first test would conduct the test at their station, then they (or another member of staff) would walk the patient to the next testing station and technician. The approaches employed varied over the course of the day. Staff interviewees indicated that when the clinic was quiet, they preferred to stay with patients 1-on-1, but when it became busier, it was more efficient for technicians to remain at one station. Most patient interviewees did not report a strong preference between these approaches (regardless of which they had experienced themselves). However, some stated that having one technician throughout made the experience more personal, offering continuity. Technicians also felt that 1-on-1 accompanying patients throughout provided a more personal experience but valued the autonomy to decide which approach was more appropriate.

#### Technology (Table 2, Section 1.4)

Clinic staff reported that technology for storing and uploading diagnostic images worked well. However, internet connectivity issues sometimes resulted in lost images, necessitating repeated appointments; accessing multiple imaging platforms to complete an assessment also added to clinician burden. However, these issues were not seen as unique to the clinic setting, but instead a common issue across NHS services. Learning and using the necessary equipment was not reported to be a problem either by technicians themselves or the senior staff that trained and supervised them.

#### Clinical staff onsite (Table 2, Section 1.5)

In this technician-led service, no clinical specialists were routinely available onsite. Patients felt sufficiently informed beforehand about the absence of doctors, though some suggested that onsite clinical staff would help address questions or unexpected complications. Some suggested alternating clinic and hospital appointments, or sharing results by telephone so that results could be discussed periodically directly with clinicians. However, no patient interviewees reported any major concerns about the absence of doctors onsite or the care they received.

### Setting of the clinic

The clinic’s novel setting (in a shopping centre, separate from existing health care services) was explored in terms of perceived accessibility, and environment within and around the clinic.

#### Accessibility (Table 2, Section 1.1)

The shopping centre has its own bus station with 14 routes serving the site and is within 15 minutes’ walk of London underground and mainline rail links. Patients’ overall experiences of the clinic were overwhelmingly positive. Several patient interviewees (6/9) noted that the journey to the clinic was longer or more complex than getting to their local hospital (this was commonly the most distant hospital from the clinic). However, no interviewees indicated that this would prevent them using the service in future. Public transport was described as time-consuming and/or complex (e.g. where there were no direct routes). Some patients chose not to drive as they believed (sometimes erroneously) that they would receive dilating eye drops, which would prevent them from driving afterwards. Some were driven by friends or family, where this was an option, and saw the ample, free parking as an advantage of the site.

Staff at a more distant referring site described dealing with daily complaints from patients with appointments booked at the clinic rather than their local hospital. These patients had usually not attended the clinic before; in some instances, they were reassured by additional information. However, some staff described having to deal with patient complaints about the journey to the clinic frequently. Some patients were reported to refuse to attend the clinic and, as such, had to be fitted into the schedule at their regular clinic.

Some patients found the clinic easily (especially if they already knew the shopping centre). However, others found the directions confusing and inadequate, recommending better communication about this, e.g. sending a shopping centre map showing the clinic location alongside the pre-appointment letter, or coloured lines on the ground, to direct patients from outside the shopping centre.

#### Environment (Table 2, Section 1.2)

Patients were positive or neutral about the shopping centre setting itself, with some noting the advantage of the location offering many distractions if appointments were delayed.

Both staff and patients commented on the clinic environment being calm and quiet. This was attributed to COVID-related social distancing measures and the low lighting inside the clinic. However, some patients initially found it difficult to adjust to the relative darkness inside the clinic compared to the bright shopping centre outside (though it should be noted that this is also a feature of hospital-based testing for these conditions).

### Staff communication and engagement

#### Communication between technicians and clinicians (Table 2, Section 3.1)

While clinic technicians were positive about the staffing model, they were less positive about the support they received from clinicians at connected hospitals. They reported that on occasions where they were uncertain about a case, support was not always accessible. Their calls were not always answered, and when they were, technicians said they frequently felt dismissed by the clinician. Clinician interviewees felt that technicians’ queries were usually appropriate, but suggested that the frustration might originate due to taking calls while seeing patients themselves, and therefore, organisation and support could be managed better, possibly through dedicated help-lines.

#### Staff engagement (Table 2, Section 3.2)

The technician-led clinic relied heavily on training, engagement, and satisfaction among those technicians. Although most technicians did not have a healthcare background, they felt the knowledge and expertise they accrued through their role were valued by senior staff. Clinic leadership worked to develop technicians by seeking regular feedback and implementing suggestions whenever possible, and by having senior clinicians give presentations on developments in the field. This helped technicians create a sense of ownership of the clinic, with most wanting to remain at the clinic and develop careers in the NHS.

#### Career progression (Table 2, Section 3.3)

A concern raised amongst clinic staff and leadership was technicians’ limited opportunities for career progression. Managers were concerned that staff might leave if they saw no route to promotion, and some staff felt unsure about their long-term prospects. As many staff joined the clinic after being laid off from other jobs due to COVID-19, the lack of progression opportunities led some to question the sustainability of a technician career.

### Future of community-based clinics (Table 2, Section 4.1)

Patients and staff, particularly clinicians, felt that technician-led testing clinics with remote specialist review were the logical model for future diagnostics and monitoring of chronic ophthalmology conditions. Such services were seen as an efficient use of time (with specialist clinicians focusing on complex, non-stable patients, and technicians supporting less complex cases, and patients spending less time in the clinic). Technicians felt the delivery model was a good alternative to traditional diagnostic services, offering patients a smoother experience overall. This view was shared by patients, while noting the importance of occasional contact with clinicians for reassurance and opportunities to ask questions.

## Discussion

Our analysis suggests that this innovative community-based diagnostic clinic was experienced positively by patients and staff as the clinic continued to develop, and services of this kind were seen as an effective way to deliver eye tests to monitor chronic eye conditions in future. Several factors influenced the delivery and experience of these services, including the organisation of eye testing processes and equipment, the shopping centre setting, and staff communication and engagement.

Delivery of eye tests in the clinic was seen as efficient, and adaptations to the configuration of testing equipment and the clinic overall were seen to enhance delivery over time; positive views of the clinic reflect previous work on the efficiency of such clinics [18]; and acceptability of the clinic setting to patients was in line with previous research [15, 16, 19]. Patients’ positive views about both the clinic and technicians suggest that patients trusted this service without the presence of clinical staff. This level of trust has been found to be important by others exploring similar service models [29]. However, in line with previous research [18], some patients indicated a preference for receiving feedback from a clinician on the day of their appointment. Patients and their friends and families valued the clinic’s shopping centre setting. However, staff and patient interviewees indicated that patients with more complex journeys to the clinic less attractive than the trip to their local hospital, though importantly patients did not indicate that this would prevent their future attendance. In addition, some patients raised concerns about the information they received in advance of the appointment. This reflects previous research indicating the importance of effective communication and engagement with patients when implementing these types of service [14]. Many technicians reported feeling trusted by senior staff and managers at the clinic; the autonomy afforded technicians around testing and their engagement in service development likely contributed to their sense of ownership of the service. This is in line with previous work indicating that trust and autonomy contribute to the development of professional identity [18, 30, 31]. Technicians’ concerns about communication with hospital-based clinicians reflect previous research on interprofessional communication in eye care services, with potential implications for team culture, service delivery, and workforce sustainability [32]. Positive feedback from patients may also help facilitate change in healthcare settings and assist in staff retention [33]. Staff engagement, including opportunities for career development, is an established influence on staff morale and organisational performance [34]; it should be noted that this issue around career development has now been addressed within the eye hospital organisation.

### Strengths and limitations

A strength of this paper is that it analysed staff and patient experiences of an innovative community-based diagnostic clinic during a period of rapid reorganisation, as the service developed during the Covid-19 pandemic. Second, integrating interview and observation data permitted a greater understanding of the factors influencing stakeholder views. Our paper has several limitations. First, while our interviewees provided a valuable range of perspectives, further research may have offered additional insights. Second, we did not interview patients who chose not to attend the clinic and therefore could not explore their reasons for this decision directly. Third, this study focused on a single diagnostic clinic located in a large urban setting, established during the pandemic; these characteristics may limit the transferability of our findings to other contexts.

### Implications for policy and services

Our findings provide important insights into experiences of an innovative community-based service. It thus contributes to the objectives set out in current NHS 10 year plan, given its focus on moving care into communities, harnessing the potential of digital innovation, and enabling greater prevention of disease [3, 8]. Both staff and patients supported future use of this clinic. These findings, taken alongside previous work on the impact of the clinic on timing of appointments [20], suggest that further development of services of this kind may be valuable, given that they may help address NHS service pressures in this context, which are longstanding and anticipated to grow [3]. Further potential advantages of such community-based services include greater opportunities for rapid development of buildings and utilities, and superior access to community pharmacy. Planners should consider how travel solutions and patient engagement might minimise concerns around travel to clinics. There may be value in offering additional communication options for sharing test results. To minimise staff turnover, career development opportunities for technicians should be explored, and have been a focus of development within the participating hospital organisation.

### Implications for future research

Mixed-method research on long-term sustainability (e.g. in terms of care delivery, patient outcomes, staff and patient experiences, and cost-effectiveness) would provide important evidence on the implications of services of this kind. For example, anecdotally, the clinic is now embedded in the hospital organisation’s clinical operations, with several issues related to communication, transport information, and technician career progression addressed, which may provide important lessons on experiences of more established services. Evidence from additional stakeholders, e.g. patients who refused to attend community-based clinics, would be valuable. Studies comparing multiple community-based clinics that use different service models in different contexts (e.g. rurality and population demographics) would provide important lessons on the organisation and delivery of such services. Finally, it is important to evaluate the impact of community services of this kind on hospital services and hospital-based clinicians who review diagnostic reports, e.g. implications for clinician burden and delivery of other clinical responsibilities.

This community-based clinic was viewed overwhelmingly positively by patients and staff and, given its established reduction of appointment delays [20], should be considered a worthwhile option for monitoring chronic eye conditions. Further optimisation and sustainability of such services may be achieved by considering patient communication (pre- and post-appointment), transport option mapping and provision of solutions, and technician career development.

## Summary

### What was known before:

- ophthalmology clinic appointment delays are a longstanding issue, with serious implications for population health, and were exacerbated by the COVID-19 pandemic.
- A community-based clinic introduced by a major eye hospital organisation in London reduced appointment delays significantly.
- While patient and staff experience of healthcare services are important factors in service delivery, sustainability, and outcomes, little is known about patient and staff experiences of clinics of this kind.

### What this study adds:

- Patients and staff experienced the clinic positively, suggesting that community-based clinics should be used more in the future.
- Patients valued shorter appointment lengths and supportive, friendly staff, and while some found the clinic harder to reach than their local hospital, they suggested this would not prevent future attendance.
- Clinic staff enjoyed working at the clinic and felt valued, whilst describing challenges in communicating with offsite clinicians and a desire for greater professional development opportunities.

## Supplementary information

Supplemental File 1 - HERCULES Consortium membership

Supplemental File 2 - Study materials - recruitment documents and topic guides

## Data Availability

The datasets generated during and/or analysed during the current study are not publicly available due conditions of interviewee anonymity agreed with the Research Ethics committee. However, relevant anonymised data are available from the corresponding author on reasonable request.
