# Supplementary material for: Patient and staff experiences of a community-based diagnostic clinic for chronic eye conditions: Qualitative analysis
Source: Eye (Lond). 2026 May 19;40(11):1746–55. doi: 10.1038/s41433-026-04528-8 (PMC13416069; doi:10.1038/s41433-026-04528-8)
Supplement: Supplementary file 1 — Supplemental File 1 - HERCULES Consortium membership [file 41433_2026_4528_MOESM1_ESM.docx]

# Collaborators – Hercules Consortium

HERCULES Consortium: (FULL LIST with affiliations available as supplementary material)

Aadil Kazi – NIHR Biomedical Research Centre, Moorfields Eye Hospital & UCL
Angus Ramsay – Research Department of Behavioural Science and Health, UCL
Anne Symons – The Bartlett School of Architecture, UCL
Connor Beddow – Moorfields Eye Hospital NHS Foundation Trust
Chris Leak - Moorfields Eye Hospital NHS Foundation Trust
Caroline S Clarke – Research Department of Primary Care and Population Health, UCL
Dun Jack Fu - NIHR Biomedical Research Centre, Moorfields Eye Hospital & UCL
Duncan Wilson – Centre for Advanced Spatial Analysis, UCL
Dhakshi Muhundhakumar - NIHR Biomedical Research Centre, Moorfields Eye Hospital & UCL
Lina Song – School of Management, UCL
Declan Flanagan - Moorfields Eye Hospital NHS Foundation Trust
Elisha Chung - Moorfields Eye Hospital NHS Foundation Trust
Ella Preston - Moorfields Eye Hospital NHS Foundation Trust
Farbod Afshar Bakeshloo – Bartlett Faculty of the Built Environment, UCL
Giovanni Ometto – Faculty of Brain Sciences, UCL
Gus Gazzard - NIHR Biomedical Research Centre, Moorfields Eye Hospital & UCL
Grant Mills – The Bartlett School of Sustainable Construction, UCL
George Damianidis - Moorfields Eye Hospital NHS Foundation Trust
Helen Baker - Moorfields Eye Hospital NHS Foundation Trust
Hari Jayaram - NIHR Biomedical Research Centre, Moorfields Eye Hospital & UCL
Ian Eames – Department of Mechanical Engineering, UCL
Iqbal Fahmi – The Bartlett School of Sustainable Construction, UCL
Irinie Roufaeel – The Bartlett School of Sustainable Construction, UCL
Jocelyn Cammack - Moorfields Eye Hospital NHS Foundation Trust
Josefine Magnusson - Institute of Epidemiology and Health Care, UCL
Jemima Unwin – Bartlett School of Environment, Energy and Resources, UCL
Jonathan Wilson - Moorfields Eye Hospital NHS Foundation Trust
Joy Adesanya - Moorfields Eye Hospital NHS Foundation Trust
Kerstin Sailer – The Bartlett School of Architecture, UCL
Kathryn Scotcher - Moorfields Eye Hospital NHS Foundation Trust
Kimberley Quan - Moorfields Eye Hospital NHS Foundation Trust
Martin Utley – Clinical Operatinal Research Unit, UCL
Matala Dyke - Moorfields Eye Hospital NHS Foundation Trust
Naomi J Fulop – Department of Applied Health Research, UCL
Natalie O'Shea - Moorfields Eye Hospital NHS Foundation Trust
Paul Foster – NIHR Biomedical Research Centre, Moorfields Eye Hospital & UCL
Peter Scully - Bartlett Faculty of the Built Environment, UCL
Paul Webster – Ubisense, Cambridge, UK
Paula Lorgelly – Department of Applied Health Research, UCL
Pei Li Ng – Department of Applied Health Research, UCL
Peter Thomas - NIHR Biomedical Research Centre, Moorfields Eye Hospital & UCL
Rachel Thompson - Moorfields Eye Hospital NHS Foundation Trust
Robin Hamilton - Moorfields Eye Hospital NHS Foundation Trust
Rosica Pachilova – Bartlett Faculty of the Built Environment, UCL
Rouba Ibrahim – School of Management, UCL
Susana Frazao Pinheiro – School of Management, UCL
Siyabonga Ndwandwe – Research Department of Primary Care and Population Health, UCL
Saheli Gandhi – The Health Care Organisation and Management Group, UCL
Samiul Alom - Moorfields Eye Hospital NHS Foundation Trust
Sherene Ettiene - Moorfields Eye Hospital NHS Foundation Trust
Sobha Sivaprasad – NIHR Biomedical Research Centre, Moorfields Eye Hospital & UCL
Stacey Angus - Moorfields Eye Hospital NHS Foundation Trust
Stephanie Kumpunen – Institute of Epidemiology and Health Care, UCL
Steve Napier – Patient Representative
Yue Tang – School of Management, UCL
Dominika Matusiak - The Bartlett School of Architecture, UCL
Ecem Ergin – The Bartlett School of Architecture, UCL
Xiaoming Li – Bartlett Faculty of the Built Environment, UCL
Muna Ayah - Moorfields Eye Hospital NHS Foundation Trust
Nadine Abdelgalil - Moorfields Eye Hospital NHS Foundation Trust
Paul Cartwright - Moorfields Eye Hospital NHS Foundation Trust
Sarah Davies - Moorfields Eye Hospital NHS Foundation Trust
Sandi Drewett - Moorfields Eye Hospital NHS Foundation Trust
Clare Feasby - Moorfields Eye Hospital NHS Foundation Trust
Simranjit Gill - Moorfields Eye Hospital NHS Foundation Trust
Steven Gill - Moorfields Eye Hospital NHS Foundation Trust
Nick Hardie - Moorfields Eye Hospital NHS Foundation Trust
Jamie Henderson - Moorfields Eye Hospital NHS Foundation Trust
Lesley Henry - Moorfields Eye Hospital NHS Foundation Trust
Peng Tee Khaw - Moorfields Eye Hospital NHS Foundation Trust
Richard Lee - Moorfields Eye Hospital NHS Foundation Trust
Sarah Martin - Moorfields Eye Hospital NHS Foundation Trust
Mary Masih - Moorfields Eye Hospital NHS Foundation Trust
Luke Nicholson - Moorfields Eye Hospital NHS Foundation Trust
Tulga Reis - Moorfields Eye Hospital NHS Foundation Trust
Nick Roberts - Moorfields Eye Hospital NHS Foundation Trust
Ana Sanchez - Moorfields Eye Hospital NHS Foundation Trust
Jon Spencer - Moorfields Eye Hospital NHS Foundation Trust
Karen Titmus - Moorfields Eye Hospital NHS Foundation Trust
Eleanor Dean – Akeso, London, UK
Nick Hynes – SOMO Global, London, UK
Olivia Jeffrey - Akeso, London, UK
Chris Robson - Akeso, London, UK
Tom Blair – Ubisense, Cambridge UK
Nick Burt – Institute of Ophthalmology, UCL
Dolores Conroy – Institute of Ophthalmology, UC
