# Supplementary material for: Patient and staff experiences of a community-based diagnostic clinic for chronic eye conditions: Qualitative analysis
Source: Eye (Lond). 2026 May 19;40(11):1746–55. doi: 10.1038/s41433-026-04528-8 (PMC13416069; doi:10.1038/s41433-026-04528-8)
Supplement: Supplementary file 2 — Supplemental File 2 - Study materials - recruitment documents and topic guides [file 41433_2026_4528_MOESM2_ESM.docx]

**Supplementary file 2 contents**

**Information sheets:**

- Patient interviews (p2)
- Non-patient interviews (p7)
- Non-participant observations (p12)

**Consent forms:**

- Patient interviews (p17)
- Non-patient interviews (p19)
- Non-participant observations (p21)

**Topic guides**

- Patient interviews (p23)
- Clinic staff (p24)
- External staff (including clinical and administrative staff) (p25)
- Senior staff (p26)

## Participant Information Sheet

**Healthcare Exemplar for Recovery from COVID-19 by Use of Linear Examination Systems**

**Project HERCULES - Qualitative Analysis of Care Delivery & Outcomes**

**(Patient Interviews)**

**Chief Investigators:**

**Professor Paul Foster, Consultant Ophthalmologist, Moorfields Eye Hospital**

**Professor Sobha Sivaprasad, Consultant Ophthalmologist, Moorfields Eye Hospital**

**Mr H Jayaram, Consultant Ophthalmologist, Moorfields Eye Hospital**

You have previously given your consent to take part in “Project HERCULES” and kindly gave permission to be approached about studies related to this project. Before you decide to participate it is important for you to understand why the research is being done and what it will involve.

Please read the following information carefully. One of our team will go through the information sheet with you and answer any questions you may have. Talk to others about the study if you wish.

**What is the purpose of this study?**

The primary aim of Project HERCULES is to develop a cost-effective, patient-centred and sustainable new model for providing NHS outpatient care in the “post pandemic era”. We plan to develop and innovate socially-distanced NHS outpatient care provided in different configurations in a retail space in Brent Cross, with aim of maximising the safety, efficiency and patient experience

We would like to speak to in detail to patients in order to understand more about their overall experience having recently attended an appointment in a “diagnostic hub”. This will enable us to incorporate this valuable feedback that will inform the development of future innovations at the new Moorfield Diagnostic Hub at Brent Cross.

**Why have I been invited to take part?**

You have been invited to take part because you have recently attended a routine outpatient follow up visit within the glaucoma or medical retina service at Moorfields Eye Hospital. You previously given your consent to take part in “Project HERCULES” and kindly gave permission to be approached about studies related to this project.

**Do I have to take part?**

It is up to you to decide if you wish to join the study. We will describe the study and go through this information sheet. If you agree to take part, we will then ask for your consent to proceed, and will document this discussion in a letter that we will send to you by post. You are free to withdraw at any time, without giving a reason. This would not affect the standard of care you receive.

**What will happen to me if I take part?**

If you agree to take part, a member of our research team will find a convenient time to speak with you on the telephone in order to discuss in more depth your recent visit to a “diagnostic hub” for your eye care. This will enable us to better understand your experiences as a patient and the events that were important for you. This will take no longer than 20 minutes.

We will ask for your permission to record the interview so that the discussion can be transcribed accurately. Any feedback or comments you provide will be completely anonymous and you will not be linked or be able to identified from the discussion that is recorded.

Analysis of the themes derived from all the interviews conducted, which will include no identifiable information, will be carried out by our colleagues at the UCL Department for Applied Health Research.

**Expenses and payments**

There will be no expenses provided for this study, as no additional visits to the hospital are necessary.

**What will I have to do?**

One of our team will go through the patient information sheet and informed consent process with you and answer any further questions you may have by telephone. Once you have had time to decide whether or not to take part, we can arrange a convenient time for the telephone interview to take place. We will send you a written summary letter of this initial telephone conversation, recording your consent to take part.

**What are the possible disadvantages and risks of taking part?**

There are no specific risks to taking part in this study.

**What are the possible benefits of taking part?**

This study will not help you directly, but the information we obtain from this study will help us to develop, evaluate and deliver a new, efficient and patient centred system of care, that will help the NHS safely and effectively look after patients with chronic eye diseases in the “post pandemic era”.

**What happens when the research study stops?**

Your participation in this study will not affect your clinical care during the study or after its completion. When the research study stops, you will continue to be followed up by your routine care team.

**What if relevant new information becomes available?**

Sometimes we get new information about the area being studied. If this happens, our team will tell you and discuss whether your participation is required in the study.

**What will happen if I don’t want to carry on with the study?**

You can withdraw from the study at any time without your clinical care being affected. Information that has already been collected may still be used but no further data will be collected from you or from your medical notes.

**What if there is a problem?**

This is very unlikely in a telephone interview-based project such as this. However, should you have any concerns you may contact the Moorfields Eye Hospital Patient Advice and Liaison Service by telephone on 020 7566 2324 or by emailing [moorfields.pals@nhs.net](mailto:moorfields.pals@nhs.net).

### **How will we use information about you?**

We will collect your name on the written summary letter we send to you confirming your consent to participate.

People will use this information to do the research or to check your records to make sure that the research is being done properly. Individuals from Moorfields Eye Hospital NHS Foundation Trust and regulatory organisations may look at your medical and research records to check the accuracy of the research study

People who do not need to know who you are will not be able to see your name, hospital number or date of birth. Your study data will have a unique participant study code number instead to ensure this is kept confidential. We will keep all information about you safe and secure.

Once we have finished the study, we will keep some of the data so we can check the results. We will write our reports in a way that no-one can work out that you took part in the study.

### **What are your choices about how your information is used?**

- You can stop being part of the study at any time, without giving a reason, but we will keep information about you that we already have.
- We need to manage your records in specific ways for the research to be reliable. This means that we won’t be able to let you see or change the data we hold about you.
- If you agree to take part in this study, you will have the option to take part in future research using your data saved from this study. If you decide that you would be happy for your anonymised study data to be used in future research please complete this section on the informed consent form.

### **Where can you find out more about how your information is used?**

You can find out more about how we use your information

- at [www.hra.nhs.uk/information-about-patients/](https://www.hra.nhs.uk/information-about-patients/)
- by asking one of the research team
- by sending an email to the Sponsor’s Data Protection Officer: email: [moorfields.ig@nhs.net](mailto:moorfields.ig@nhs.net)

Moorfields Eye Hospital NHS Foundation Trust is the sponsor for this study which is based only in the United Kingdom. We will be using information from your medical records in order to undertake the overall study and will act as the data controller for this study. This means that we are responsible for looking after your information and using it properly. Moorfields Eye Hospital NHS Foundation Trust will keep identifiable information about you for 5 years after the study has finished.

**What will happen to the results of the research study?**

The results of the study will be presented at national and international conferences and published in scientific journals. All data will be anonymous and none of the participants involved the study can be identified from any reports published. We will also share the results of this study with NHS England, the National Institute for Health Research, Moorfields Eye Charity, the Macular Society and Glaucoma UK. Should you wish to see the results of the study in the future, please let one of our team know.

**Who is organising and funding the research?**

This project is being funded through support from the National Institute for Health Research Biomedical Research Centre at Moorfields Eye Hopsital and the UCL Institute of Ophthalmology, and commercial partners Zeiss, Optos and Ubisense. The project is sponsored by Moorfields Eye Hospital NHS Foundation Trust.

**Who has reviewed the study?**

All research in the NHS is looked at by an independent group of people called a Research Ethics Committee, to protect your interests. This study has been reviewed and given favourable opinion by the North East-York Research Ethics Committee (REC reference 21/NE/0164) on 17th August 2021.

**Further information and contact details**

You have the right to ask questions concerning your participation in this study at any time. You may ask questions before you sign the consent, at any time during your participation in the study, and after you are finished with the study.

The people to contact for any questions or problems during the study are listed below:

Project HERCULES Operational Manager: XXXX, Telephone: XXXX / Email: XXXX

If you have any concerns regarding the conduct of the study, or wish to speak to someone independent of the research team, please contact the Patient Advice and Liaison Service (PALS) on 020 7566 2325.

Thank you for taking the time for reading the study details in this Participant Information Sheet.

## Participant Information Sheet

**Healthcare Exemplar for Recovery from COVID-19 by Use of Linear Examination Systems**

**Project HERCULES - Qualitative Analysis of Care Delivery & Outcomes**

**(Non-Patient Stakeholder Interviews)**

**Chief Investigators:**

**Professor Paul Foster, Consultant Ophthalmologist, Moorfields Eye Hospital**

**Professor Sobha Sivaprasad, Consultant Ophthalmologist, Moorfields Eye Hospital**

**Mr H Jayaram, Consultant Ophthalmologist, Moorfields Eye Hospital**

You are being invited to take part in this research study because you are a significant professional stakeholder in the development and implementation of diagnostic hubs which aim to deliver NHS care for chronic eye diseases. Before you decide to participate it is important for you to understand why the research is being done and what it will involve.

Please read the following information carefully. One of our team will go through the information sheet with you and answer any questions you may have. Talk to others about the study if you wish.

**What is the purpose of this study?**

The primary aim of Project HERCULES is to develop a cost-effective, patient-centred and sustainable new model for providing NHS outpatient care in the “post pandemic era”. We plan to develop and innovate socially-distanced NHS outpatient care provided in different configurations in a retail space in Brent Cross, with aim of maximising the safety, efficiency and patient experience

We would like to speak to patients and various stakeholders who have been involved in the design and implementation of diagnostic hubs (service leaders/managers, clinical staff members, diagnostic hub staff, design partners, commissioners/national bodies) in order to understand more about their opinions on the development, delivery and impact of diagnostic hubs. This will enable us to incorporate this valuable feedback that will help inform the development of future innovations at the new Moorfield Diagnostic Hub at Brent Cross.

**Why have I been invited to take part?**

You have been invited to take part because you are a significant professional stakeholder in the development and implementation of diagnostic hubs, which aim to deliver NHS care for chronic eye diseases.

**Do I have to take part?**

It is up to you to decide if you wish to join the study. We will describe the study and go through this information sheet. If you agree to take part, we will then ask for your consent to proceed, and will document this discussion in a letter that we will send to you by post or email. You are free to withdraw at any time, without giving a reason.

**What will happen to me if I take part?**

If you agree to take part, a member of our research team will find a convenient time to speak in more depth with you on the telephone in order to discuss your opinions on the implementation, operations and perceived impact of diagnostic hubs. This will take no longer than 20 minutes.

We would ask for your permission to record the interview so that the discussion can be transcribed accurately. Any feedback or comments you provide will be completely anonymous and you will not be linked or be able to identified from the discussion.

Analysis of the themes derived from all the interviews conducted, which will include no identifiable information, will be carried out by our colleagues at the UCL Department for Applied Health Research.

**Expenses and payments**

There will be no expenses provided for this study, as no additional visits to the hospital are necessary.

**What will I have to do?**

One of our team will go through the patient information sheet with you and answer any further questions you may have by telephone. Once you have had time to decide whether or not to take part, we can arrange a convenient time for the telephone interview to take place. We will send you a written summary letter of this initial telephone conversation, recording your consent to take part.

**What are the possible disadvantages and risks of taking part?**

There are no specific risks to taking part in this study.

**What are the possible benefits of taking part?**

This study will not help you directly, but the information we obtain from this study will help us to develop, evaluate and deliver a new, efficient and patient centred system of care, that will help the NHS safely and effectively look after patients with chronic eye diseases in the “post pandemic era”.

**What will happen if I don’t want to carry on with the study?**

You can withdraw from the study at any time. Information that has already been collected may still be used.

**What if there is a problem?**

This is very unlikely in a telephone interview-based project such as this. However, should you have any concerns you may contact the Moorfields Eye Hospital Patient Advice and Liaison Service by telephone on 020 7566 2324 or by emailing [moorfields.pals@nhs.net](mailto:moorfields.pals@nhs.net).

### **How will we use information about you?**

We will collect your name on your completed study consent form.

People will use this information to do the research or to check your records to make sure that the research is being done properly. Individuals from Moorfields Eye Hospital NHS Foundation Trust and regulatory organisations may look at the study records to check the accuracy of the research study

People who do not need to know who you are will not be able to see your name. Your study data will have a unique participant study code number instead to ensure this is kept confidential. We will keep all information about you safe and secure.

Once we have finished the study, we will keep some of the data so we can check the results. We will write our reports in a way that no-one can work out that you took part in the study.

### **What are your choices about how your information is used?**

- You can stop being part of the study at any time, without giving a reason, but we will keep information about you that we already have.
- We need to manage your records in specific ways for the research to be reliable. This means that we won’t be able to let you see or change the data we hold about you.
- If you agree to take part in this study, you will have the option to take part in future research using your data saved from this study. If you decide that you would be happy for your anonymised study data to be used in future research please complete this section on the informed consent form.

### **Where can you find out more about how your information is used?**

You can find out more about how we use your information

- at [www.hra.nhs.uk/information-about-patients/](https://www.hra.nhs.uk/information-about-patients/)
- by asking one of the research team
- by sending an email to the Sponsor’s Data Protection Officer: email: [moorfields.ig@nhs.net](mailto:moorfields.ig@nhs.net)

Moorfields Eye Hospital NHS Foundation Trust is the sponsor for this study which is based only in the United Kingdom, and will act as the data controller for this study. This means that we are responsible for looking after your information and using it properly. Moorfields Eye Hospital NHS Foundation Trust will keep identifiable information about you for 5 years after the study has finished.

**What will happen to the results of the research study?**

The results of the study will be presented at national and international conferences and published in scientific journals. All data will be anonymous and none of the participants involved the study can be identified from any reports published. We will also share the results of this study with NHS England, the National Institute for Health Research, Moorfields Eye Charity, the Macular Society and Glaucoma UK. Should you wish to see the results of the study in the future, please let one of our team know.

**Who is organising and funding the research?**

This project is being funded through support from the National Institute for Health Research Biomedical Research Centre at Moorfields Eye Hopsital and the UCL Institute of Ophthalmology, and commercial partners Zeiss, Optos and Ubisense. The project is sponsored by Moorfields Eye Hospital NHS Foundation Trust.

**Who has reviewed the study?**

All research in the NHS is looked at by an independent group of people called a Research Ethics Committee, to protect your interests. This study has been reviewed and given favourable opinion by the North East-York Research Ethics Committee (REC reference 21/NE/0164) on 17th August 2021.

**Further information and contact details**

You have the right to ask questions concerning your participation in this study at any time. You may ask questions before you sign the consent, at any time during your participation in the study, and after you are finished with the study.

The people to contact for any questions or problems during the study are listed below:

Project HERCULES Operational Manager: XXXX, Telephone: XXXX / Email: XXXX

If you have any concerns regarding the conduct of the study, or wish to speak to someone independent of the research team, please contact the Patient Advice and Liaison Service (PALS) on 020 7566 2325.

Thank you for taking the time for reading the study details in this Participant Information Sheet.

## Participant Information Sheet

**Healthcare Exemplar for Recovery from COVID-19 by Use of Linear Examination Systems**

**Project HERCULES - Qualitative Analysis of Care Delivery & Outcomes**

**(Non-Patient Stakeholder Interviews)**

**Chief Investigators:**

**Professor Paul Foster, Consultant Ophthalmologist, Moorfields Eye Hospital**

**Professor Sobha Sivaprasad, Consultant Ophthalmologist, Moorfields Eye Hospital**

**Mr H Jayaram, Consultant Ophthalmologist, Moorfields Eye Hospital**

You are being invited to take part in this research study because you are a significant professional stakeholder in the development and implementation of diagnostic hubs which aim to deliver NHS care for chronic eye diseases. Before you decide to participate it is important for you to understand why the research is being done and what it will involve.

Please read the following information carefully. One of our team will go through the information sheet with you and answer any questions you may have. Talk to others about the study if you wish.

**What is the purpose of this study?**

The primary aim of Project HERCULES is to develop a cost-effective, patient-centred and sustainable new model for providing NHS outpatient care in the “post pandemic era”. We plan to develop and innovate socially-distanced NHS outpatient care provided in different configurations in a retail space in Brent Cross, with aim of maximising the safety, efficiency and patient experience

We would like to speak to patients and various stakeholders who have been involved in the design and implementation of diagnostic hubs (service leaders/managers, clinical staff members, diagnostic hub staff, design partners, commissioners/national bodies) in order to understand more about their opinions on the development, delivery and impact of diagnostic hubs. This will enable us to incorporate this valuable feedback that will help inform the development of future innovations at the new Moorfield Diagnostic Hub at Brent Cross.

**Why have I been invited to take part?**

You have been invited to take part because you are a significant professional stakeholder in the development and implementation of diagnostic hubs, which aim to deliver NHS care for chronic eye diseases.

**Do I have to take part?**

It is up to you to decide if you wish to join the study. We will describe the study and go through this information sheet. If you agree to take part, we will then ask for your consent to proceed, and will document this discussion in a letter that we will send to you by post or email. You are free to withdraw at any time, without giving a reason.

**What will happen to me if I take part?**

If you agree to take part, a member of our research team will find a convenient time to speak in more depth with you on the telephone in order to discuss your opinions on the implementation, operations and perceived impact of diagnostic hubs. This will take no longer than 20 minutes.

We would ask for your permission to record the interview so that the discussion can be transcribed accurately. Any feedback or comments you provide will be completely anonymous and you will not be linked or be able to identified from the discussion.

Analysis of the themes derived from all the interviews conducted, which will include no identifiable information, will be carried out by our colleagues at the UCL Department for Applied Health Research.

**Expenses and payments**

There will be no expenses provided for this study, as no additional visits to the hospital are necessary.

**What will I have to do?**

One of our team will go through the patient information sheet with you and answer any further questions you may have by telephone. Once you have had time to decide whether or not to take part, we can arrange a convenient time for the telephone interview to take place. We will send you a written summary letter of this initial telephone conversation, recording your consent to take part.

**What are the possible disadvantages and risks of taking part?**

There are no specific risks to taking part in this study.

**What are the possible benefits of taking part?**

This study will not help you directly, but the information we obtain from this study will help us to develop, evaluate and deliver a new, efficient and patient centred system of care, that will help the NHS safely and effectively look after patients with chronic eye diseases in the “post pandemic era”.

**What will happen if I don’t want to carry on with the study?**

You can withdraw from the study at any time. Information that has already been collected may still be used.

**What if there is a problem?**

This is very unlikely in a telephone interview-based project such as this. However, should you have any concerns you may contact the Moorfields Eye Hospital Patient Advice and Liaison Service by telephone on 020 7566 2324 or by emailing [moorfields.pals@nhs.net](mailto:moorfields.pals@nhs.net).

### **How will we use information about you?**

We will collect your name on your completed study consent form.

People will use this information to do the research or to check your records to make sure that the research is being done properly. Individuals from Moorfields Eye Hospital NHS Foundation Trust and regulatory organisations may look at the study records to check the accuracy of the research study

People who do not need to know who you are will not be able to see your name. Your study data will have a unique participant study code number instead to ensure this is kept confidential. We will keep all information about you safe and secure.

Once we have finished the study, we will keep some of the data so we can check the results. We will write our reports in a way that no-one can work out that you took part in the study.

### **What are your choices about how your information is used?**

- You can stop being part of the study at any time, without giving a reason, but we will keep information about you that we already have.
- We need to manage your records in specific ways for the research to be reliable. This means that we won’t be able to let you see or change the data we hold about you.
- If you agree to take part in this study, you will have the option to take part in future research using your data saved from this study. If you decide that you would be happy for your anonymised study data to be used in future research please complete this section on the informed consent form.

### **Where can you find out more about how your information is used?**

You can find out more about how we use your information

- at [www.hra.nhs.uk/information-about-patients/](https://www.hra.nhs.uk/information-about-patients/)
- by asking one of the research team
- by sending an email to the Sponsor’s Data Protection Officer: email: [moorfields.ig@nhs.net](mailto:moorfields.ig@nhs.net)

Moorfields Eye Hospital NHS Foundation Trust is the sponsor for this study which is based only in the United Kingdom, and will act as the data controller for this study. This means that we are responsible for looking after your information and using it properly. Moorfields Eye Hospital NHS Foundation Trust will keep identifiable information about you for 5 years after the study has finished.

**What will happen to the results of the research study?**

The results of the study will be presented at national and international conferences and published in scientific journals. All data will be anonymous and none of the participants involved the study can be identified from any reports published. We will also share the results of this study with NHS England, the National Institute for Health Research, Moorfields Eye Charity, the Macular Society and Glaucoma UK. Should you wish to see the results of the study in the future, please let one of our team know.

**Who is organising and funding the research?**

This project is being funded through support from the National Institute for Health Research Biomedical Research Centre at Moorfields Eye Hopsital and the UCL Institute of Ophthalmology, and commercial partners Zeiss, Optos and Ubisense. The project is sponsored by Moorfields Eye Hospital NHS Foundation Trust.

**Who has reviewed the study?**

All research in the NHS is looked at by an independent group of people called a Research Ethics Committee, to protect your interests. This study has been reviewed and given favourable opinion by the North East-York Research Ethics Committee (REC reference 21/NE/0164) on 17th August 2021.

**Further information and contact details**

You have the right to ask questions concerning your participation in this study at any time. You may ask questions before you sign the consent, at any time during your participation in the study, and after you are finished with the study.

The people to contact for any questions or problems during the study are listed below:

Project HERCULES Operational Manager: XXXX, Telephone: XXXX / Email: XXXX

If you have any concerns regarding the conduct of the study, or wish to speak to someone independent of the research team, please contact the Patient Advice and Liaison Service (PALS) on 020 7566 2325.

Thank you for taking the time for reading the study details in this Participant Information Sheet.

Date:

Dear

Further to the telephone conversation on xx/xx/xxxx, thank you for agreeing to participate by telephone for the following study:

**“Project HERCULES - Qualitative Analysis of Care Delivery & Outcomes”**

**(Patient Interviews)**

Please find enclosed a copy of the study Participant Information Sheet

Version: 1.0 Date: 29/07/2021

As discussed with our research team, we can confirm that you are eligible for the study and during the telephone conversation you agreed to the following:

- To take part in a brief telephone interview to discuss in depth your recent visit to a “diagnostic hub” for your eye care.
- To give permission for the interview to be recorded so that it can be transcribed accurately for subsequent analysis.
- Participation in the study is voluntary and you are free to withdraw at any time without giving any reason without your medical care or legal rights being affected.
- Relevant sections of data collected during the study may be looked at by Regulatory Authorities or from the NHS Trust where it is relevant to your taking part in research. You have given permission for these individuals to have access.
- Information will be shared anonymously with other researchers at the UCL Department of Applied Health Research for subsequent analysis.
- You have agreed to take part in this study.

If you do not agree with any of the above statements, please contact the study team as soon as possible using the contact details provided below.

If you have any further questions please do not hesitate to contact us.

Yours sincerely

**Professor Paul Foster**

**Professor Sobha Sivaprasad**

**Dr Hari Jayaram**

Telephone: 020 7253 3411 / Email: [moorfields.hercules@nhs.net](mailto:moorfields.hercules@nhs.net)

Date:

Dear

Further to the telephone conversation on xx/xx/xxxx, thank you for agreeing to participate by telephone for the following study:

**“Project HERCULES - Qualitative Analysis of Care Delivery & Outcomes” (Non-Patient Stakeholder Interviews)**

Please find enclosed a copy of the study Participant Information Sheet

Version: 1.0 Date: 29/07/2021

As discussed with our research team, we can confirm that you are eligible for the study and during the telephone conversation you agreed to the following:

- To take part in a brief telephone interview to discuss your opinions on the implementation, operations and perceived impact of diagnostic hubs.
- To give permission for the interview to be recorded so that it can be transcribed accurately for subsequent analysis.
- Participation in the study is voluntary and you are free to withdraw at any time without giving any reason without your legal rights being affected.
- Relevant sections of data collected during the study may be looked at by Regulatory Authorities or from the NHS Trust where it is relevant to your taking part in research. You have given permission for these individuals to have access.
- Information will be shared anonymously with other researchers at the UCL Department of Applied Health Research for subsequent analysis.
- You have agreed to take part in this study.

If you do not agree with any of the above statements, please contact the study team as soon as possible using the contact details provided below.

If you have any further questions please do not hesitate to contact us.

Yours sincerely

**Professor Paul Foster**

**Professor Sobha Sivaprasad**

**Dr Hari Jayaram**

Telephone: 020 7253 3411 / Email: [moorfields.hercules@nhs.net](mailto:moorfields.hercules@nhs.net)

Participant Unique Study Identification Number:

R

G

**CONSENT FORM**

**Healthcare Exemplar for Recovery from COVID-19 by Use of Linear Examination Systems**

**Project HERCULES - Qualitative Analysis of Care Delivery & Outcomes**

**(Non-Participant Observations – Hub Staff)**

*Please initial*

*each box*

1. I confirm that I have read the information sheet dated 29/07/21 (version 1.0) for the above study. I have had the opportunity to consider the information, ask questions and have had these answered satisfactorily.
2. I understand that my participation is voluntary and that I am free to withdraw at any time without giving any reason, without my employment or legal

rights being affected.

1. I understand that observations of my interactions with patients will be

documented by the research team for the purposes of this study.

1. I understand that relevant sections of the data collected

during the study may be looked at by individuals from regulatory authorities or from Moorfields Eye Hospital NHS Foundation Trust, where it is relevant to my taking part in this research. I give permission for these individuals to have access to this relevant data.

1. I understand that the data collected will be shared with research collaborators from University College London, and that it will not be possible to identify me from these records.
2. I agree to take part in the above study.

__________

Name of Participant Date Signature

__________ ___

Name of Person Taking Consent Date Signature

**Topic guide: patients**

**Setup**

- Confirm purpose of research; run through PIS and consent
- Confirm status (age, condition, hub attended)

***Please tell me about your recent visit to a diagnostic hub for your eye assessment – the experiences and events which were important for you.***

**Firstly, how were you communicated with before your visit?**

- How did you hear about the hub/service and your appointment?
- For how long have you been going to prior service (e.g. hospital)?
- Enough information to know what would happen/where to go? anything that could have helped more?
- Confident beforehand about going to the hub?

**What happened when you first arrived at the diagnostic hub/service?**

- How did you find getting to the hub/service? Able to get there independently or have to rely on friend/ relative?
- Meeting staff? Who were they?
- Get necessary information? Explanations?

**How did you find going through your different tests?**

- Going through different tests – how did they together? delays, waiting?
- Overall burden in going through the battery of tests?
- Any problems (e.g. technology, route-finding around environment)?
- Did the environment feel safe and supportive? e.g. access to toilets, COVID safety
- What made a difference – help or hindrance?
- Any different/better/worse than previous appointments?

**How did you feel about the staff in the hub?**

- confident they knew what they were doing?
- enough information about what was happening?
- treated with respect/dignity?
- how was it different from seeing a doctor at hospital?

**What happened after your assessments at the hub?**

- ending the visit – did you know what would come next?
- how did you feel about having to wait for results? what could have helped?
- How was your follow-up – e.g. getting results

**Any lessons or reflections on your experience?**

- Did you find this a useful way to get your assessment?
- Anything that really helped or could be done better?
- Would you want to go back to this hub again? Would you recommend it to friends?

***Finally, is there anything else you would you like to add?***

**Topic guide: clinic staff (including technicians and administrative staff)**

**Setup**

- Confirm purpose of research; run through PIS and consent

**Background**

- What is your role? How long have you been doing this? Previous role/career?
- How have you been working at the diagnostic hubs (all iterations?)?

**How did you first become aware of the diagnostic hub?**

- What information, who shared it?
- What did you think of the idea?
- Did you or your colleagues have any concerns?

**What was done to introduce the new hub?**

- Planning – how were the hubs developed?
- Implementation – how were they put into action?
- Facilitation – what support was there for implementation?
- Governance – how were services overseen?
- Leadership – who led development of the services?
- Staffing/new roles – what roles were required? recruitment?
- How did you find training for this role (e.g. buddying, assessment)?
- How was shift between different iterations? any adaptations?

**How is the hub working at present?**

- What is the current process followed by patients at the hub?
- What were the differences between iterations? – preference, reasons?
- Working in a new environment?
- Technical factors (tests or connection with remote clinicians)?
- Interacting with patients (meeting needs/addressing problems)?
- Any factors that make a difference?

**What has been the impact of the hub?**

- Thinking about differences between different iterations …
  - Probe how differences may be down to layout v increased experience
- For patients – timely care, quality of care, experience, outcomes, burden?
  - how do they feel about not seeing a doctor?
- For services – effectiveness, efficiency, quality?
- For you personally? personal benefits/problems?

**Any lessons or reflections on your experience?**

- What has worked well/badly?
  - Probe how this may be down to service v increased experience
- Anything you think should be done differently?
- What might make a difference
- Do you think this service should continue in future?
- Would you continue working here – next steps?
  - For staff recruited from other industries: How has working here influenced your view of career in health care/ NHS?

***Finally, is there anything else you would you like to add?***

**Topic guide: external staff (including clinical and administrative staff)**

**Setup**

- Confirm purpose of research; run through PIS and consent

**Background**

- What is your role? How long have you been doing this?

**How did you first become aware of the diagnostic hub?**

- What information, who shared it?
- What did you think of the idea?
- Did you or your colleagues have any concerns?

**What was done to introduce the new hub?**

- What (if any) discussions were held with you about sending patients to the hub?
- Implementation – how was triaging put into action?
- Facilitation – what support was there for implementation?
- Governance – how were services overseen?
- Leadership – who led development of the services?

**How has the process been working since implementation of the hub?**

- What is the current process for triaging patients?
  - How has this changed since initial implementation (if at all)?
- Technical factors (tests or connection with remote clinicians)?
- Feedback, if any, from patients (meeting needs/addressing problems)?
- (Any factors that make a difference?)

**What has been the impact of the hub?**

- For patients – timely care, quality of care, experience, outcomes, burden?
  - how do they feel about not seeing a doctor?
- For services – effectiveness, efficiency, quality?
  - How has this changed over time?
- For the team/ ophthalmology services within [location] overall?
  - How has this changed over time?
- For you personally? personal benefits/problems?
  - How has this changed over time?

**Any lessons or reflections on your experience?**

- What has worked well/badly?
- Anything you think should be done differently?
- What might make a difference
- Do you think this service should continue in future?

***Finally, is there anything else you would you like to add?***

**Topic guide: Senior staff**

**Setup**

- Confirm purpose of research; run through PIS and consent

**Background**

- What is your role? How long have you been doing this?
- How have you been involved in introducing diagnostic hubs?

**What was the background to the diagnostic hub?**

- What are the drivers for diagnostic hubs? (services, service/system leadership, funding, policy)
- What evidence/learning influenced its development?
- What did you think of the idea? Did you or your colleagues have concerns?

**Role of diagnostic hub**

- Moorfields have an established model of hub-based diagnostics; how do you see the role of hubs?
  - What are the benefits/ drawbacks?
  - How (if at all) is Brent Cross different?
- How do you see your own role within the establishment of diagnostic hubs?
- What do trusts need to consider if wanting to follow this model?

**What was done to introduce the new hub?**

- Planning – how were the hubs developed?
- Implementation – how were they put into action?
- Facilitation – what support was there for implementation?
- Governance – how were services overseen?
- Leadership – who led development of the services?

**What has been the impact of the hub?**

- For patients – timely care, quality of care, experience, outcomes, burden?
- For services – effectiveness, efficiency, quality?
- For you personally? personal benefits/problems?

**Moving beyond the pilot phase - business as usual/wider implementation**

- How might hubs of this kind be implemented in other settings – e.g. smaller environments, more rural areas, etc?

**Any lessons or reflections on your experience?**

- What has worked well/badly?
- Anything you think should be done differently?
- What might make a difference?
- What might be some of the barriers to this model being implemented more widely?

***Finally, is there anything else you would you like to add?***
